# miR-16-5p, miR-21-5p, and miR-155-5p in circulating vesicles as psoriasis biomarkers

**DOI:** 10.1038/s41598-025-91532-9

**Published:** 2025-02-26

**Authors:** Carlos A. Guzmán-Martín, Rogelio F. Jiménez-Ortega, María Fernanda Ortega-Springall, Mario Peña-Peña, Ana Elena Guerrero-Ponce, María Elisa Vega-Memije, Luis M. Amezcua-Guerra, Fausto Sánchez-Muñoz, Rashidi Springall

**Affiliations:** 1 Doctorado en Ciencias Biológicas y de la Salud, Universidad Autónoma Metropolitana, Mexico city, Mexico; 2 Departamento de Inmunología, Instituto Nacional de Cardiología Ignacio Chávez, Mexico City, Mexico; 3 Laboratorio de Genómica del Metabolismo Óseo, Instituto Nacional de Medicina Genómica, Mexico City, Mexico; 4 Departamento de Ciencias de la Acupuntura, Universidad Estatal del Valle de Ecatepec, Ecatepec de Morelos, Mexico; 5 Departamento de Dermatología, Hospital General Dr. Manuel Gea González, Mexico City, Mexico; 6 Hospital General de México Dr. Eduardo Liceaga, Mexico City, Mexico

**Keywords:** Bioinformatics, MicroRNAs, Extracellular vesicles, Microarrays, Biomarkers, Psoriasis, PASI, Psoriasis area severity index, Molecular biology, Biomarkers

## Abstract

Psoriasis is a chronic skin disorder marked by fast skin cell growth, leading to thick, red, scaly patches. MicroRNAs are small, non-coding RNA molecules that play a crucial role in post-transcriptional gene regulation. This study investigates miR-16-5p, miR-21-5p, and miR-155-5p expression in psoriasis EVs and assesses their biomarker potential, exploring associated target genes and pathways via bioinformatics. A cross-sectional and case-control study included 40 psoriasis patients, with blood samples collected in EDTA tubes. RNA from extracellular vesicles was isolated using Qiagen kits, and miRNAs were quantified via RT-qPCR. Bioinformatic analysis predicted target genes using databases like miRDB and TargetScan. Gene expression data from GEO was processed, and differentially expressed genes were identified. This study assessed miR-16-5p, miR-21-5p, and miR-155-5p expression in psoriasis patients’ circulating vesicles versus controls, finding significantly lower levels in patients. ROC analysis confirmed their diagnostic potential. A positive correlation of miR-16-5p with the Psoriasis Area Severity Index (PASI) suggests severity marker potential. Bioinformatics identified 378 common dysregulated genes, revealing key pathways and gene interactions in psoriasis. A heat map confirmed miRNA-mediated gene suppression in the disease. This study identifies miR-16-5p, miR-21-5p, and miR-155-5p as potential psoriasis biomarkers, in addition to finding significant gene interactions and pathways involved in psoriasis pathophysiology.

**Supplementary Information:**

The online version contains supplementary material available at 10.1038/s41598-025-91532-9.

## Introduction

Psoriasis is a chronic inflammatory skin disease characterized by fast skin cell growth that results in thick, red, and scaly patches^1^. The prevalence of psoriasis varies across different populations; however it is estimated to affect approximately 2–3% of the global population. Psoriasis can develop at any age, but it most commonly appears between 15 and 35 years old^1^. While the exact cause of psoriasis is not fully understood, the pathogenesis involves a complex interplay between genetic predisposition and environmental triggers, leading to dysregulation of the immune system and hyperproliferation of keratinocytes^2,3^. Despite advances in understanding its pathophysiology, the exact mechanisms underlying psoriasis remain incompletely elucidated. In recent years, there has been growing interest in the role of non-coding RNAs in the development and progression of psoriasis^4,5^. In this sense, microRNAs (miRNAs) are small, non-coding RNA molecules with an approximate of 22 nucleotides in length that play a crucial role in post-transcriptional gene regulation. They modulate gene expression by binding to specific messenger RNA (mRNA) molecules, thereby inhibiting their translation or promoting their degradation^6^. MiRNAs have been found to be involved in various cellular processes, including proliferation, differentiation, apoptosis, and immune response, and in addition, have been studied as biomarkers in diverse pathological scenarios^7,8^. In the context of psoriasis, dysregulation of miRNAs has been observed in both lesional skin and peripheral blood cells of affected individuals. These altered miRNA expression patterns are thought to contribute to the abnormal immune responses and excessive proliferation of skin cells characteristic of the psoriatic lesions. Several miRNAs have been identified as critical players in psoriasis pathogenesis. For instance, miR-16, involved in cell cycle regulation and apoptosis, is often dysregulated in psoriasis, affecting keratinocyte proliferation and differentiation^9,10^. MiR-21, frequently upregulated in inflammatory diseases including psoriasis, promotes inflammation by targeting anti-inflammatory genes^11^. MiR-155, a critical regulator of immune responses, is upregulated in psoriasis, modulating genes involved in inflammatory signaling and T-cell activation^12^. Dysregulation of these miRNAs can disrupt the normal process of skin cell turnover, leading to hyperproliferation and abnormal differentiation described in psoriasis.

While miRNAs have been widely studied in the context of psoriasis, the role of miR-16-5p, miR-21-5p, and miR-155-5p within circulating extracellular vesicles (EVs) remains largely unexplored. Extracellular vesicles are membrane-bound particles secreted by various cell types, playing a crucial role in intercellular communication by transporting proteins, lipids, and RNA molecules, including miRNAs. EVs offer a unique advantage as they protect their molecular cargo from degradation, allowing miRNAs to remain stable in circulation^13^. By encapsulating miRNAs, EVs facilitate the long-range transport of regulatory molecules, potentially mediating systemic immune responses and inflammation, key features of psoriasis^14^. Understanding the miRNA content of EVs may reveal novel biomarkers and therapeutic targets, providing deeper insight into psoriasis pathophysiology.

Therefore, this study aims to investigate the expression levels of miR-16-5p, miR-21-5p, and miR-155-5p in circulating EVs from psoriasis patients, assessing their potential as biomarkers for the disease. Additionally, we will explore the target genes and pathways associated with these miRNAs through comprehensive bioinformatics analyses.

## Results

We studied a total of 40 patients with psoriasis. Their median age was 53 years old (IQR 45.2 to 58.7 years), and almost half (42.5%) were female. The median body mass index (BMI) was 29.8 kg/m2. Nearly half (47.5%) of the patients were obese. Other common health problems included dyslipidemia (25%), diabetes mellitus (20%), hypertension (22.5%), and smoking (27.5%). Detailed information is provided in Table 1. The control group consisted of 43 healthy individuals with a median age of 53 years (IQR: 46–59), of whom 46% were female (*n* = 20). The median BMI was 27.2 kg/m^2^ (IQR: 24.2–30.1). The controls were selected to match the psoriasis group in terms of age and gender distribution to ensure comparability between the two groups.

**Table 1:** Characterization of study participants.

Variable	Psoriasis patients
Age in years, median (IQR)	53 (45.2–58.7)
Female sex^a^	17 (42.5)
Body mass index, median (IQR)	29.8 (24.7–33.3)
Obesity^a^	19(47.5)
Dyslipidemia^a^	10(25)
Diabetes mellitus^a^	8(20)
Hypertension^a^	9(22.5)
Tabaquism^a^	11(27.5)
PASI, median (IQR)	3.3(1.7–5.8)
*N* = 40	

^a^n (%); n, number of participants.

**Fig. 1:**
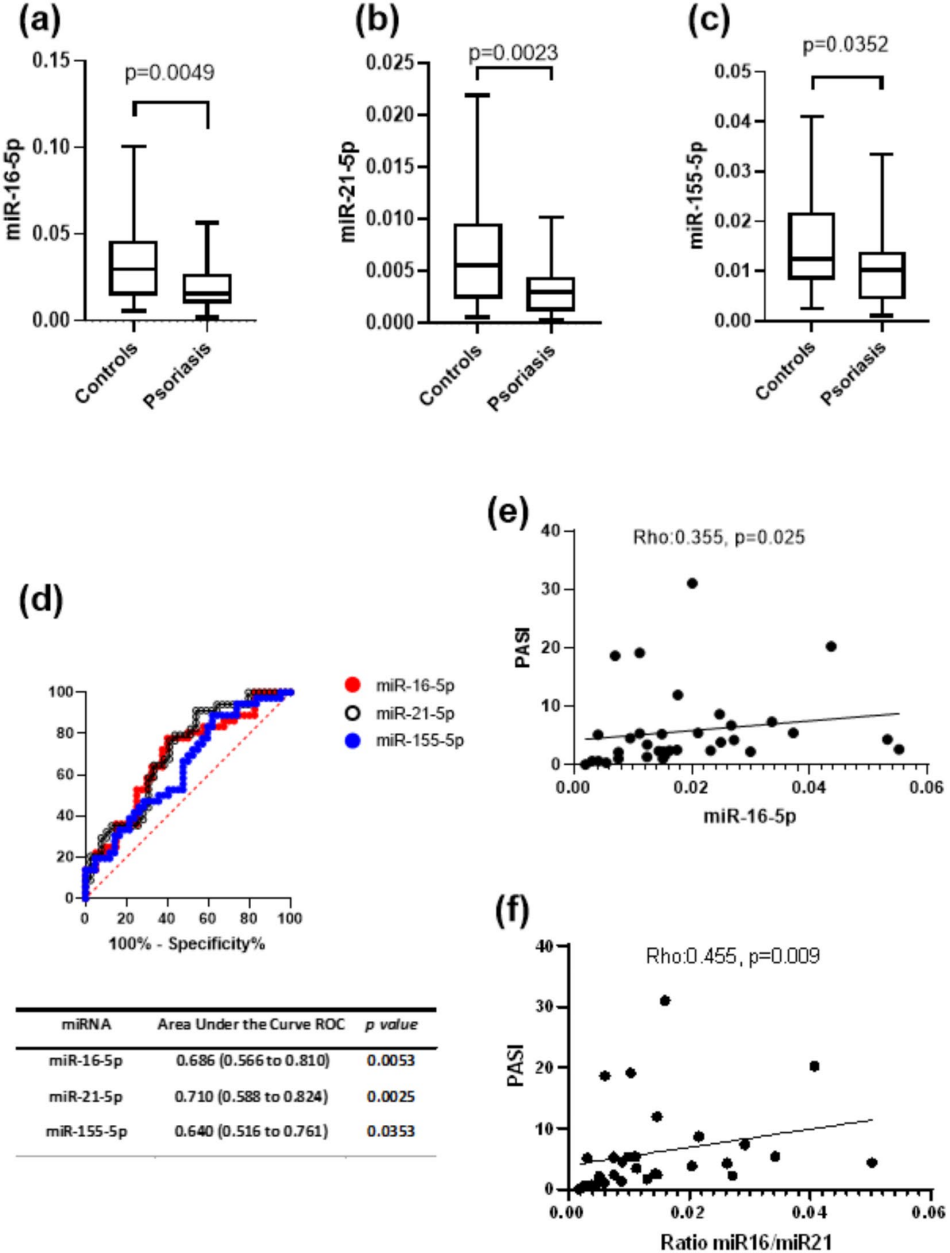
microRNA expression, discriminative value and correlation analysis (**a**–**c**) Lower expression levels of miR-16-5p, miR-21-5p, and miR-155-5p in the psoriasis group compared to controls. (**d**) Area under the curve ROC analysis to test the discriminative value of studied miRNAs. (**e**–**f**) Positive correlation of miR-16-5p and ratio miR-16-5p/miR21 with Psoriasis Area Severity Index PASI.

In the expression analysis, we found lower expression levels of miR-16-5p, miR-21-5p, and miR155 between psoriasis patients compared with the control group, miR-16-5p: median 0.0158, IQR: 0.0099 to 0.0270 v*s* median 0.0299, IQR: 0.0147 to 0.0462, p-value: 0.0049. miR-21-5p: median 0.0030, IQR: 0.0011 to 0.0045 v*s* median 0.0056, IQR: 0.0024 to 0.0096, p-value: 0.0023. miR-155-5p: median 0.0102, IQR: 0.0043 to 0.0140 v*s* median 0.0125, IQR: 0.0083 to 0.0218, p-value: 0.0352, (Fig. 1a-c). In addition, we performed an Area Under the Curve ROC analysis. Interestingly, we found that the studied miRNAs are capable to discriminate between psoriasis patients from controls: miR-16-5p: AUC: 0.6861, 95% CI: 0.5664 to 0.8058; miR-21-5p: AUC: 0.7059, 95% CI: 0.5876 to 0.8242; miR-150: AUC: 0.6389, 95% CI: 0.5164 to 0.7614, (Fig. 1d). Subsequently, the Spearman test showed a significant positive correlation between the PASI index and expression levels of miR-16-5p, Rho: 0.355, p-value: 0.025 and between PASI and miR-16-5p miR-21-5p Ratio, Rho: 0.455, *p* = 0.009 Fig. 1e and (Fig. 1f).

**Fig. 2:**
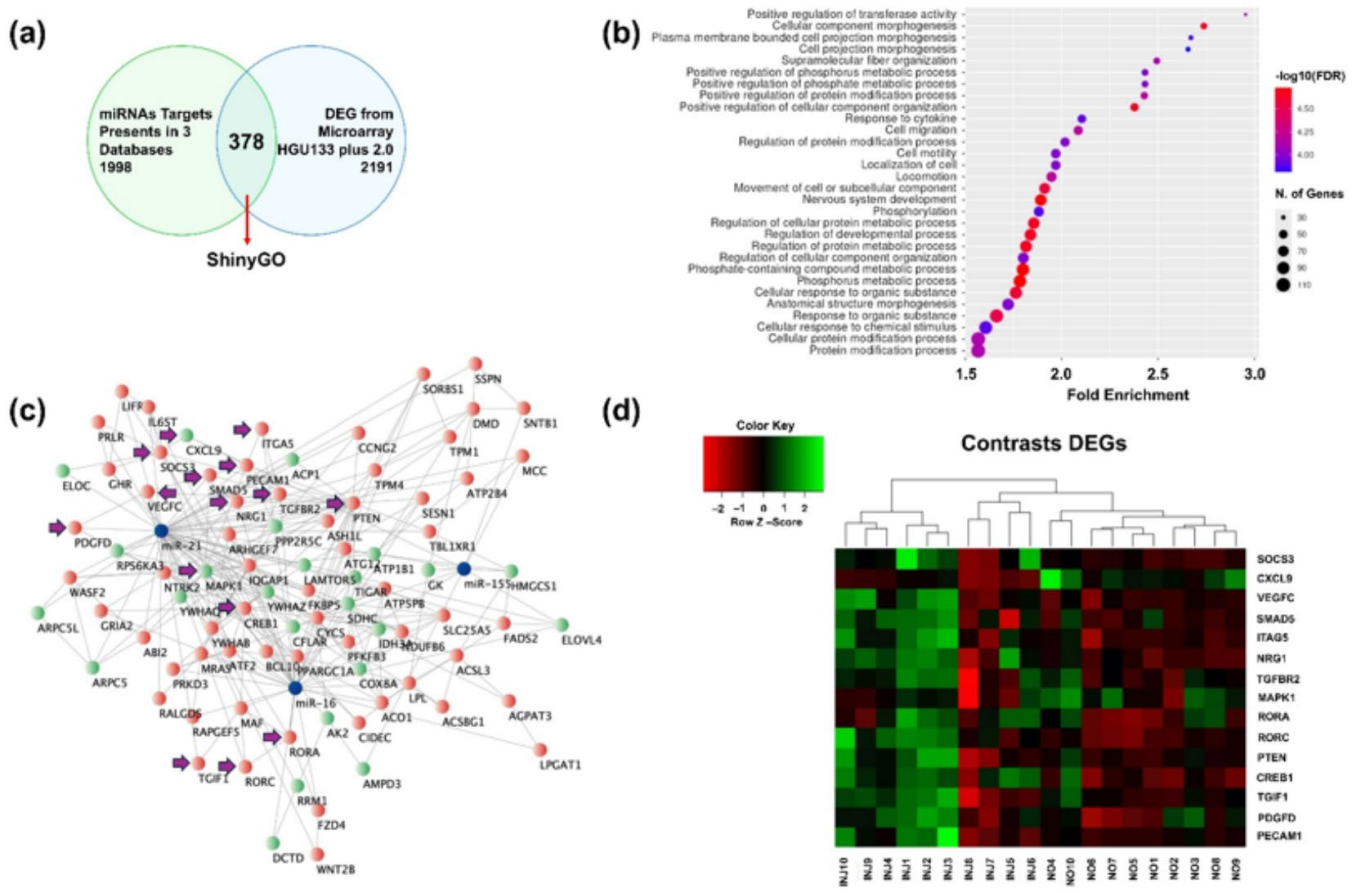
Bioinformatic analysis. Integrated analysis of psoriasis-associated mechanisms (**a**) Venn diagram depicting common dysregulated genes identified through bioinformatic analysis. (**b**) Enrichment analysis results highlighting key pathways implicated in psoriasis pathogenesis. Biological processes involved in the development of psoriasis (**c**) miRNAs-genes interaction network illustrating regulatory relationships between miRNAs (miR-16-5p, miR-21-5p, miR-155-5p) and dysregulated genes. Down-regulated genes are shown in red, up-regulated genes in green, and miRNAs in blue. Arrows show selected genes involved in the development of psoriasis. (**d**) Heatmap visualizing the expression levels of identified target genes across psoriasis patients (INJ) and healthy controls (NO), showcasing patterns of miRNA-mediated gene suppression.

Furthermore, we conducted a bioinformatic analysis of three datasets containing dysregulated genes from psoriatic skin biopsies compared to controls. This data was then compared with genes associated with miR-16-5p, miR-21-5p, and miR-155-5p, as identified from the prediction databases described in the methods. Interestingly, we identified 378 genes common between both analyses (Fig. 2a). From this set of genes, 227 downregulated genes were identified and 151 upregulated genes (supplementary Table 1). These results could be due to different factors such as the geographic region, the lifestyle of the patients, their metabolic status, and other confounding variables that could modify the genetic expression in the analyzed samples. Interestingly, a set of genes could be identified that, despite the effects of multiple variables, are consistent in patients with psoriasis. Using these shared dysregulated genes, we performed an enrichment analysis, revealing different biological processes and signaling pathways linked to the pathophysiology of psoriasis (Fig. 2b and supplementary figure). Then, we identified the primary genes associated with hsa-miR-16-5p, hsa-miR-21-5p, and hsa-miR-155-5p from these pathways and constructed an interaction network (Fig. 2c).

Finally, based on an extensive bibliographic review, we identified 15 differentially expressed genes that interact with specific signaling pathways and are targets of the miRNAs studied in psoriasis. Higher miRNA levels correlated with lower expression levels of these target genes. A heat map was created using microarray data to visualize differential expression patterns between psoriatic and control samples, highlighting miRNA-mediated gene suppression (Fig. 2d). The results support the hypothesis of miRNA-mediated suppression in psoriasis pathophysiology.

## Discussion

In this study, we aimed to explore the expression levels of miR-16-5p, miR-21-5p, and miR-155-5p in psoriasis patients and their potential role in the disease’s pathophysiology. Our findings indicate that these miRNAs are significantly downregulated in psoriasis patients compared to healthy controls. Moreover, the diagnostic potential of these miRNAs and their correlation with the Psoriasis Area and Severity Index suggest their relevance in disease progression and severity. Additionally, through bioinformatics analysis, we identified 378 dysregulated genes shared across psoriasis patients, offering valuable insights into key pathways and gene interactions involved in the pathogenesis of the disease.

Our study consisted of 40 psoriasis patients with a median age of 53 years. The high prevalence of comorbid conditions such as obesity, dyslipidemia, diabetes mellitus, hypertension, and smoking align with previous studies reporting these conditions as common among psoriasis patients^15–17^. These comorbidities are known to exacerbate psoriasis symptoms and complicate treatment, highlighting the need of integrated healthcare approaches for these patients.

On the other hand, the significant downregulation of miR-16-5p, miR-21-5p, and miR-155-5p in psoriasis patients compared to controls is a notable finding. MiR-16 is known for its role in cell cycle regulation and apoptosis^18^. Its reduced expression in psoriasis patients may contribute to the hyperproliferative nature of psoriatic skin lesions^19^. Additionally, miR-16 potentially regulates DUSP7 expression, and discrepancies between mRNA and protein expression have been noted due to miRNA regulation and transcription-translation synchronization^9^. The regulation of DUSP7 by miR-16 is significant because DUSPs (dual-specificity phosphatases) play a crucial role in modulating the MAPK (mitogen-activated protein kinase) signaling pathway, which is central to the pathogenesis of psoriasis^20,21^. MiR-21 is a well-documented oncomiR involved in various inflammatory pathways and immune responses^22^. Its downregulation in psoriasis could indicate a complex regulatory mechanism where miR-21 might play a role in mitigating inflammation, contrary to its pro-inflammatory roles in other diseases^23^. Studies have shown that inhibiting miR-21 in activated T cells increases apoptosis, suggesting that miR-21 suppresses apoptosis and contributes to psoriatic skin inflammation^24^. Finally, the potential of miR-21-5p as a therapeutic target and biomarker in the context of psoriasis is under investigation^25,26^.

MiR-155 is another critical player in immune regulation and inflammatory responses^27^. Its lower levels in psoriasis patients suggest that miR-155 might be involved in the suppression of excessive immune responses, potentially acting as a compensatory mechanism to counteract the chronic inflammation seen in psoriasis^28–30^. Our findings indicate a downregulation of miR-16-5p, miR-21-5p, and miR-155-5p in circulating extracellular vesicles from psoriasis patients, which contrasts with reports such as those by Alatas et al., who observed upregulation of miR-155-5p in whole blood. This discrepancy may be attributed to the distinct biological compartments analyzed: EVs, which are enriched in miRNAs involved in cell-to-cell communication, versus the bulk miRNA content in whole blood^19^.

The observation of decreased miRNA levels in circulating EVs, despite hypothesizing active trafficking from inflamed skin, may be explained by the selective and dynamic nature of miRNA sorting into EVs, as highlighted in several studies. Specifically, miRNA sorting is influenced by factors such as the availability of miRNA target transcripts in producer cells and the intracellular transcriptomic environment, as demonstrated by Squadrito et al.^31^. For instance, in the context of inflammation, cytokines like IL-4 can induce significant transcriptomic changes, potentially altering miRNA sorting dynamics. This selective packaging could result in the depletion of specific miRNAs in EVs, even if overall trafficking increases. Additionally, Ozawa et al. suggest that lower expression levels of certain miRNAs in EVs may correlate with more pathological scenarios in breast cancer patients. In the case of inflamed skin, local tissue damage or chronic inflammation might alter the expression and availability of miRNAs in cells, reducing their incorporation into EVs despite active trafficking^32^. This could reflect a pathological state where the mechanisms driving miRNA packaging into EVs are disrupted or overridden by other cellular processes. Therefore, the observed decrease in circulating miRNA levels in EVs could be the result of both selective sorting and pathological downregulation of miRNAs at the site of inflammation. These mechanisms may act concurrently, leading to the apparent paradox of reduced circulating EV miRNAs despite active trafficking from inflamed tissues. A deeper mechanistic investigation such as profiling EV cargo in severe psoriatic patients or functional assays to assess miRNA-target interactions would be a valuable next step.

Previous studies and our bioinformatic analysis suggest that these miRNAs play an active role in suppressing target genes in psoriatic skin. It is possible that miRNAs are selectively packaged into circulating EVs as part of a compensatory mechanism to mitigate local inflammation in psoriatic lesions by exporting these regulatory molecules into the bloodstream. This could explain the observed reduction in circulating miRNA levels while maintaining their regulatory functions at the tissue level. Further research comparing miRNA expressions across multiple biological matrices, including whole blood and EVs, is necessary to provide a more comprehensive understanding of miR-155-5p and other miRNAs’ roles in psoriasis.

The ROC analysis demonstrated that miR-16-5p, miR-21-5p, and miR-155-5p could discriminate between psoriasis patients and healthy controls, with AUC values indicating moderate diagnostic accuracy. MiR-21-5p showed the highest diagnostic potential with an AUC of 0.7059. These findings suggest that these miRNAs could serve as non-invasive biomarkers for psoriasis diagnosis and monitoring^33^. The moderate AUC values highlight the need for further validation in larger cohorts and the potential for combining these miRNAs with other biomarkers to enhance diagnostic accuracy.

The positive correlation between the PASI scores and the expression levels of miR-16-5p, as well as the miR-16-5p/miR-21-5p ratio, underscores the clinical relevance of these miRNAs. PASI is a widely used tool to assess the severity and extent of psoriasis, and diverse studies have correlated miRNAs expression with disease activity^34,35^. Our findings also support the potential use of these miRNAs as biomarkers for monitoring disease progression and response to therapy. However, one limitation of our study is the relatively small number of patients stratified across severity groups, with the majority of our cohort presenting mild psoriasis. This uneven distribution may have restricted our ability to detect significant differences in miRNA expression between the mild, moderate, and severe groups.

Our bioinformatic analysis identified 378 genes dysregulated in psoriatic skin biopsies that are potential targets of miR-16-5p, miR-21-5p, and miR-155-5p. Several authors have investigated the possible use of a bioinformatic approach to elucidate molecular interactions in psoriasis pathophysiology^36,37^. Enrichment analysis of these genes revealed several signaling pathways implicated in psoriasis pathophysiology, including the TNF, and PI3K-Akt pathways^38,39^. These pathways are known to be involved in inflammation, cell proliferation, and survival, which are critical processes in the development and maintenance of psoriatic lesions^40,41^. The interaction network constructed from these pathways further elucidated the complex regulatory mechanisms at play. Genes identified in this network, such as TNF, IL-6, and STAT3, are well-known mediators of inflammation and immune responses in psoriasis^42^. The downregulation of these target genes in the context of elevated miRNA levels supports the hypothesis of miRNA-mediated suppression of pro-inflammatory and pro-proliferative genes, which could be a compensatory mechanism to limit the chronic inflammation characteristic of psoriasis.

Based on our bibliographic review, we selected 15 differentially expressed genes that interact with specific signaling pathways and are targets of the miRNAs studied. The heat map created from microarray data clearly showed the differential expression patterns of these genes between psoriatic and control samples. The observed downregulation of these genes in the presence of elevated miRNA levels provides strong evidence for miRNA-mediated suppression in psoriasis.

This finding aligns with the growing body of literature that supports the role of miRNAs in regulating gene expression post-transcriptionally. In psoriasis, the overexpression of certain miRNAs can lead to the suppression of key regulatory genes involved in maintaining skin homeostasis and immune responses. This dysregulation contributes to the pathological features of psoriasis, including hyperproliferation of keratinocytes and chronic inflammation.

## Clinical implications and future directions

The identification of miR-16-5p, miR-21-5p, and miR-155-5p as potential biomarkers for psoriasis offers significant clinical implications. Extracellular vesicles were chosen for this study due to their role in transporting signaling molecules, including miRNAs, which reflect dynamic changes in disease states like psoriasis. EV-derived miRNAs may provide greater specificity as biomarkers, as they are selectively packaged and actively involved in intercellular communication. Although this study did not compare miRNA abundance between whole blood and EVs, future research should explore this to assess whether EVs enhance diagnostic sensitivity and specificity. These miRNAs could serve as valuable diagnostic tools to complement existing criteria, offering more precise and earlier diagnosis. Monitoring miRNA levels may also help assess disease severity and predict exacerbations, improving patient management and treatment outcomes. Additionally, therapeutic strategies targeting these miRNAs, such as mimics or inhibitors, could be explored to restore normal gene expression and mitigate psoriasis pathology. Future research should validate these findings in larger, diverse cohorts, conduct longitudinal studies to examine miRNA changes during treatment and disease progression, and use experimental techniques like qRT-PCR or RNA-seq to confirm the involvement of dysregulated genes in psoriasis pathogenesis.

## Conclusions

In conclusion, our study demonstrates dysregulated expressions of miR-16-5p, miR-21-5p, and miR-155-5p in circulating vesicles of psoriasis patients, highlighting their potential as diagnostic biomarkers for the disease. The ROC analysis underscores their discriminative value, while the positive correlation between miR-16-5p expression levels and PASI suggests a role in disease severity assessment.

Furthermore, our bioinformatic analysis revealed differential expressions of pathways associated with psoriasis and an interaction network implicating miR-16-5p, miR-21-5p, and miR-155-5p. These findings provide valuable insights into the molecular mechanisms underlying psoriasis pathogenesis and underscore the importance of microRNAs in regulating pathways involved in the disease.

## Methods

### Patients

A cross-sectional and case-control study with a bioinformatic approach was conducted involving 40 patients diagnosed with psoriasis from the dermatology outpatient clinic at Hospital General Dr. Manuel Gea Gonzalez in Mexico City, Mexico along with 43 healthy controls. Blood samples of five mL were collected in EDTA tubes from all participants. Clinical data, including the assessment of disease activity using the Psoriasis Area and Severity Index, were extracted from each participant’s medical record at the time of enrollment. Prior to blood sampling, all participants provided informed consent in accordance with international guidelines, good clinical practice, and the Helsinki Declaration. The protocol was reviewed and approved by the internal committee of the Instituto Nacional de Cardiología Ignacio Chávez, under registration number 20-1170.

### RNA isolation of extracellular vesicles and MiRNAs quantification

Extracellular vesicles were isolated from 600 µL of plasma participants via centrifugation at 10,000 × g for 30 min at 4 °C to eliminate cell debris. The resulting supernatant was recovered. For RNA isolation from EVs, the exoRNeasy serum/plasma midi kit from Qiagen (Hilden, Germany) was used. During RNA purification, a standardized amount of cel-miR-39 spike-in control from Qiagen was included, following the manufacturer’s guidelines. The RNA extracted from EVs was promptly converted into cDNA for further analysis.

For miRNA determination, a two-step RT-qPCR approach was employed using RT-primer specific assays and TaqMan probes for miR-16-5p (Assay ID 000391, Catalogue number 4427975), miR-21-5p (Assay ID 000397, Catologue number 4427975), and miR-155-5p (Assay ID 002623, Catalogue number 4427975) (Applied Biosystems; CA, USA). Each RT reaction used 1.5 µL from the 14 µL eluted RNA obtained through the TaqMan MicroRNA Reverse Transcription Kit (Applied Biosystems; CA, USA). The RT reaction and PCR cycling conditions following the manufacturer’s instructions. Relative concentrations of miRNAs were normalized using Ct values of cel-miR-39, and the values were calculated using the 2 − ΔΔCt method. Ct values for cel-miR-39 ranged from 20 to 22 cycles for both total plasma and EVs RNA isolations.

## Bioinformatic analysis

### MiRNAs target genes prediction

To predict potential target genes for miRNAs hsa-miR-16-5p, hsa-miR-21-5p, and hsa-miR-155-5p, we utilized multiple computational algorithms. These algorithms identify target genes by matching the seed region of a miRNA (nucleotides 2–7) with the 3’ untranslated region (3’UTR) of its mRNA. We employed several databases and tools, including TargetScan, which evaluates the accessibility of target gene binding sites, and miRanda, which filters binding sites based on miRNA thermodynamic properties. Additional software applications incorporated machine learning techniques that use biological data parameterization and other predicted features. The databases used in this analysis were:

- miRDB v6.0 (http://mirdb.org/miRDB/).
- miRWalk v3.0 (http://mirwalk.umm.uni-heidelberg.de/).
- TargetScan v7.2 (http://www.targetscan.org/vert_80/).
- PITA v5.0.0 (https://tools4mirs.org/software/target_prediction/pita/).

Target genes identified by at least three databases were selected. The predicted target genes for each miRNA were then compiled into a single list for subsequent comparative analysis.

### Expression microarray analysis

To uncover genes associated with psoriasis that might be targeted by hsa-miR-16-5p, hsa-miR-21-5p, and hsa-miR-155-5p, we delved into existing literature. Our search focused on studies that explored changes in genetic expression profiles linked to psoriasis in humans at the genomic level, and we specifically sought data that was publicly available. For this purpose, we turned to the Gene Expression Omnibus (GEOs) platform (https://www.ncbi.nlm.nih.gov/geo/). We identified one relevant study, GSE14905^41^, which analyzed changes in gene expression profiles of skin biopsies from patients with psoriasis compared to control groups. In this study, the original files in CEL format were retrieved from the HGU133 plus 2.0 microarray of the Affymetrix platform. For each study, the original files in CEL format were recovered from the HGU133 plus 2.0 microarray of the Affymetrix platform. We selected 10 control and 10 psoriasis samples that exhibited the least variability during exploratory analysis. Samples were labeled as normal (NO) for controls and psoriatic injury (INJ) for psoriasis-affected samples. The selection of these specific samples was based on their stability, ensuring that outliers or highly variable samples were excluded to minimize bias.

To further validate the sample size, we applied the coefficient of variation (CV) method recommended by Affymetrix, which compares the CV values across different numbers of replicates at various percentiles of signal intensities (25th, 50th, and 75th percentiles). We determined that the CV plateaued at seven replicates for each percentile, indicating that additional samples were unlikely to significantly improve accuracy in estimating standard deviations. Thus, the chosen sample size of 10 replicates per group provided sufficient statistical power to detect differentially expressed genes while maintaining accuracy and reproducibility of the findings^42^.

### Data processing and differentially expressed genes (DEGs)

Differentially expressed genes (DEGs) were analyzed using microarray data from GSE14905, processed with the “robust multiarray average” (RMA) method in the R environment. The “Affy” package was used for probe-level transformation, background correction, and normalization. DEGs were identified using logFC thresholds (<-0.5 and > 0.5) and an p value < 0.05. Overlapping genes between datasets were visualized using a Venn diagram, identifying consistently dysregulated genes across studies. This integrative approach provided insights into psoriasis pathophysiology while prioritizing the robust data from GSE14905.

### Enrichment analysis

The shared genes were analyzed using ShinyGO Enrichment Analysis + more software v0.741 (http://bioinformatics.sdstate.edu/go74/) for Homo sapiens species to extract enrichment pathways from the KEGG database. This analysis revealed correlations among the enriched pathways, leading to the creation of an interaction network highlighting the most significant biological processes. Additionally, the STRING-KEGG Pathway tool (https://string-db.org/) was employed to identify and select signaling pathways associated with psoriasis pathophysiology. This selected group of pathways was used to construct an interaction network linking hsa-miR-16-5p, hsa-miR-21-5p, and hsa-miR-155-5p with their target genes, facilitated by Cytoscape v3.9.1 software.

### Identification of potential candidate genes

Finally, through a comprehensive literature review, we evaluated the intricate relationship between target genes and signaling pathways relevant to psoriasis. This thorough analysis led us to identify 15 differentially expressed genes that not only interact with specific signaling pathways, but also serve as targets of the miRNAs analyzed. Our investigation also indicated the possibility of a downward trend in their expression levels, consistent with a proof of concept that elevated miRNA expression might correspond to reduced expression of their target genes. Subsequently, to further explore these dynamics, we generated a heat map. This heat map was instrumental in examining the expression levels of the target genes on the microarray arrays evaluated, as shown in Fig. 2d.

### Statistical analysis

Statistical analysis was carried out through the Statistical Package for Social Sciences (SPSS v26). The Shapiro-Wilk test was conducted to assess the distribution of quantitative variables. Regarding descriptive statistics, the median and interquartile ranges are shown for quantitative variables, and qualitative variables are presented as frequencies and percentages. For comparison of miRNAs between cases and controls, the Mann-Whitney U test was performed. The diagnostic potential of miR-16-5p, miR-21-5p, and miR-155-5p was evaluated using receiver operating characteristic (ROC) curve analysis. The area under the curve (AUC) values were calculated to assess their discriminative capacity between psoriasis patients and controls. Metrics including sensitivity, specificity, and the 95% confidence intervals. Correlation analysis was carried out using the Spearman method. A p-value less than 0.05 was considered statistically significant.

## Electronic supplementary material

Below is the link to the electronic supplementary material.

Supplementary Material 1

Supplementary Material 2

## Data Availability

Availability of Data and Materials: The datasets analyzed in this study are derived from previously published studies and are available in the Gene Expression Omnibus (GEO) repository. The relevant accession numbers are provided throughout the manuscript. No new datasets were generated for this study. However, the raw data for the miRNA expression analysis can be shared upon reasonable request to the corresponding authors.
