# Supplementary material for: miR-16-5p, miR-21-5p, and miR-155-5p in circulating vesicles as psoriasis biomarkers
Source: Sci Rep. 2025 Feb 26;15:6971. doi: 10.1038/s41598-025-91532-9 (PMC11865590; doi:10.1038/s41598-025-91532-9)
Supplement: Supplementary file 2 — Supplementary Material 2 [file 41598_2025_91532_MOESM2_ESM.pdf]

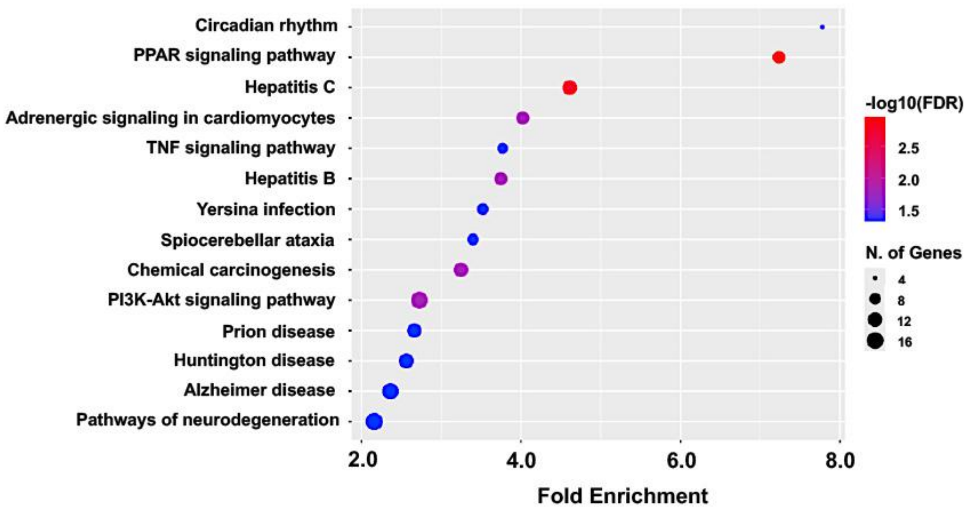

Supplementary figure 1. Pathway enrichment analysis of differentially expressed genes. The x-axis represents fold enrichment, while the y-axis lists the significantly enriched pathways. Red dot color represents higher significance. Dot size corresponds to the number of genes involved in each pathway.
